# Choriocarcinoma-Driven Uterine Perforation in a Bicornuate Anatomy: An Elusive Encounter

**DOI:** 10.7759/cureus.48841

**Published:** 2023-11-15

**Authors:** Maoulik Kumar P Modi, Garima A Jasani, Nitin S Raithatha, Pokhraj P Suthar

**Affiliations:** 1 Radiology, Parul Institute of Medical Sciences and Research, Vadodara, IND; 2 Radiology, Citi Neuro Centre, Hyderabad, IND; 3 Obstetrics and Gynaecology, Pramukhswami Medical College, Bhaikaka University, Karamsad, IND; 4 Diagnostic Radiology and Nuclear Medicine, Rush University Medical Center, Chicago, USA

**Keywords:** mri, ultrasound, uterine perforation, choriocarcinoma, bicornuate uterus

## Abstract

Choriocarcinoma, an aggressive gestational trophoblastic disease, infrequently manifests with spontaneous uterine perforation. We report the case of a 22-year-old female with five months of amenorrhea presenting with acute abdominal pain. Ultrasound and MRI assessment highlighted a uterine perforation with choriocarcinoma. Subsequent total abdominal hysterectomy revealed choriocarcinoma in the bicornuate uterus with uterine perforation. Histopathological analysis confirmed the diagnosis of choriocarcinoma in the cornu of the uterus. Timely diagnosis is vital to reduce mortality. Notably, choriocarcinoma in a bicornuate uterus is exceptionally rare. Radiological evaluations are critical for diagnosis, staging, and follow-up.

## Introduction

Gestational trophoblastic disease (GTD) encompasses a spectrum of conditions with varying levels of malignancy [1]. Among these, choriocarcinoma is the most aggressive variant due to its rapid growth and high potential for spreading to other parts of the body [2]. The incidence of choriocarcinoma varies significantly worldwide. For instance, in the United States, it is relatively rare, occurring in only two out of every 100,000 pregnancies, while in China, the rate is much higher at 202 per 100,000 pregnancies [3]. This report focuses on a rare case where choriocarcinoma presented in a bicornuate uterus, resulting in uterine perforation. It is important to note that choriocarcinoma is an uncommon cause of spontaneous uterine perforation, and having a bicornuate uterus or prior molar pregnancy is a known risk factor [4]. Additionally, the preoperative radiological findings and the subsequent follow-up of the present case were discussed.

## Case presentation

**Figure 1:**
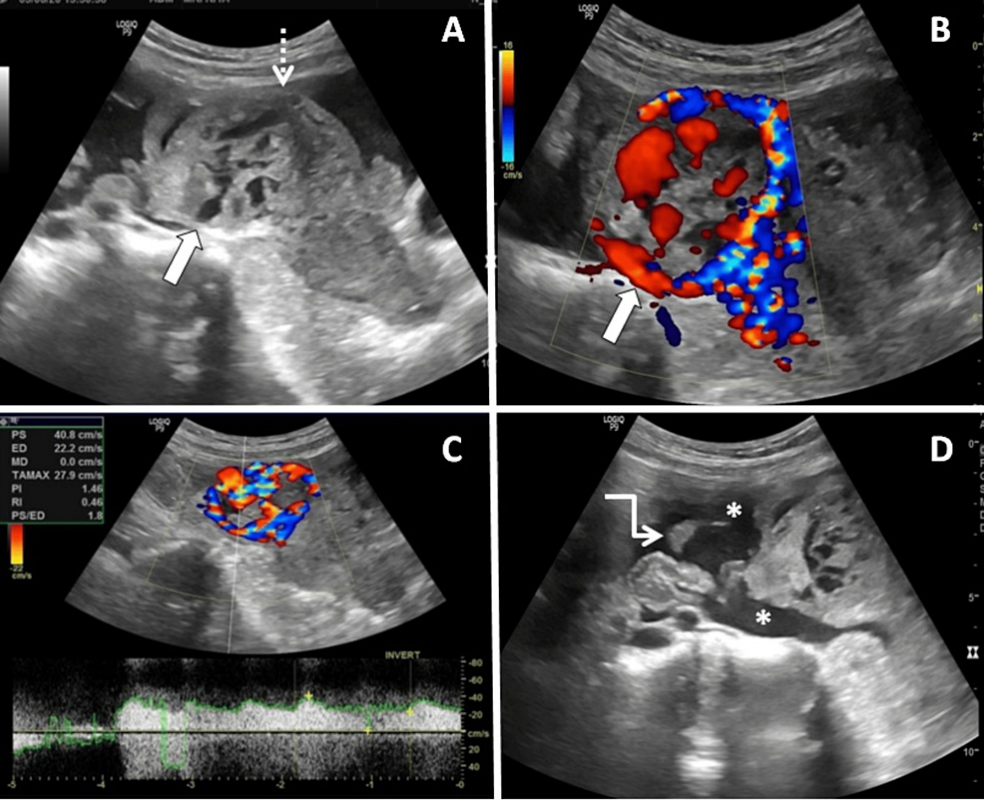
USG of the pelvis with color Doppler reveals an enlarged uterus with an irregular outline. The lesion exhibits heterogeneous echogenicity and anechoic areas within the endometrial cavity, and there is infiltration of the fundal myometrium (solid white arrow in A). Thinning of the overlying myometrium is evident, with a suspicious rent (dashed white arrow in A). The lesion demonstrates markedly increased vascularity (solid white arrow in B) with a low-resistance flow on color Doppler, having a resistive index of 0.46 (as seen in C). Additionally, omental thickening is evident (white elbow arrow in D), along with hemoperitoneum (white asterisk in D).

A 22-year-old gravida 3 patient with a five-month history of amenorrhea presented to the emergency department with symptoms of breathlessness, vomiting, diarrhea, and abdominal pain lasting for six hours. Her obstetric record noted one prior molar pregnancy and one live birth. Initial laboratory investigations showed a reduced hemoglobin level at 6.4 mg/dl, an elevated total leukocyte count of 34,000 cells/ul, and a serum β-hCG (beta-human chorionic gonadotropin) level surpassing 100,000 IU. Pelvic ultrasonography revealed an enlarged uterus with a heterogeneous echotexture and an irregular contour of the fundus. An indistinct, ill-defined lesion, manifesting heterogeneous echogenicity interspersed with anechoic regions, was discerned within the endometrial cavity and myometrium. The overlying myometrium was markedly thinned out with a suspicious area of rent. Notably, heightened vascularity was associated with the lesion, with spectral waveform analyses identifying a low-resistance waveform. Further observations included omental thickening and suggestive signs of hemoperitoneum (Figure 1). Based on these imaging presentations, an initial diagnosis of either a complete invasive mole or choriocarcinoma was proposed. A contrast-enhanced magnetic resonance imaging (MRI) of the pelvis was advised for further clarity.

**Figure 2:**
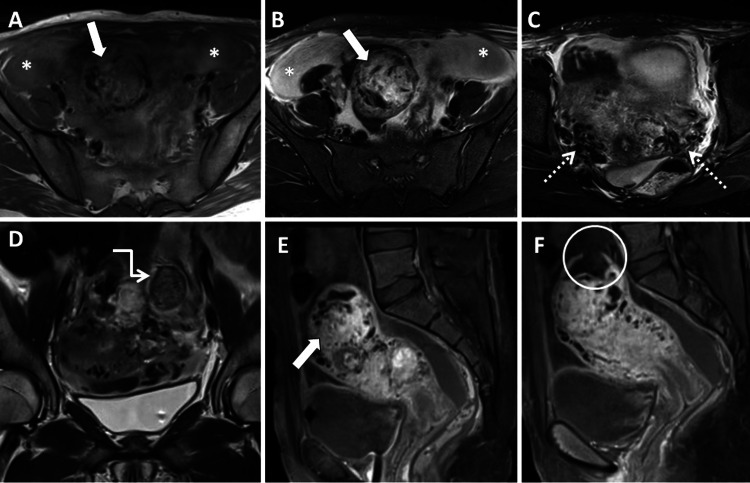
MRI of the pelvis with and without contrast. (A) T1-weighted axial, (B and C) T2-weighted fat-saturated axial, (D) T2-weighted coronal, and (E and F) sagittal T1 post-contrast images demonstrate a lesion in the fundus of the uterus that is T1 iso- to mildly hyperintense and T2 heterogeneously hyperintense. This lesion involves the myometrium and the superior-most portion of the endometrium (solid white arrows in A and B). Multiple areas of flow voids are seen within the lesion, adjacent to the lesion, in the remaining uterine myometrium, in the parametrium, and in the parauterine region of the pelvis (open white arrows in C). Also noteworthy is the enlarged left external iliac lymph node (white elbow arrow in D) and the hemoperitoneum (white asterisk in A and B). Post-contrast MRI images show intense, heterogeneous post-contrast enhancement of the lesion (solid white arrow in E) along with irregularity in the uterine outline and a discontinuity of the fundal myometrium due to uterine perforation (open white circle in F).

The pelvis MRI highlighted an irregular lesion within the endometrial region of the uterine fundus, which seemed to infiltrate the adjacent myometrium. This lesion demonstrated significant heterogeneous enhancement, appearing iso- to mildly hyperintense on T1WI and heterogeneously hyperintense on T2WI. The presence of multiple flow voids signaled tortuous and dilated vascular pathways. Additional flow voids near the lesion extended into the surrounding uterine myometrium, parametrium, and para-uterine pelvic area. These characteristics suggested either a completely invasive hydatiform mole or choriocarcinoma. Furthermore, irregularities in the uterine outline and a notable contour bulge over the fundus and the posterior-left lateral aspect of the uterine body, accompanied by a tear in the myometrium's superior fundal portion, hinted at uterine perforation (Figure 2). Enlargements in the bilateral external iliac lymph nodes and the presence of significant pelvic fluid were also observed.

**Figure 3:**
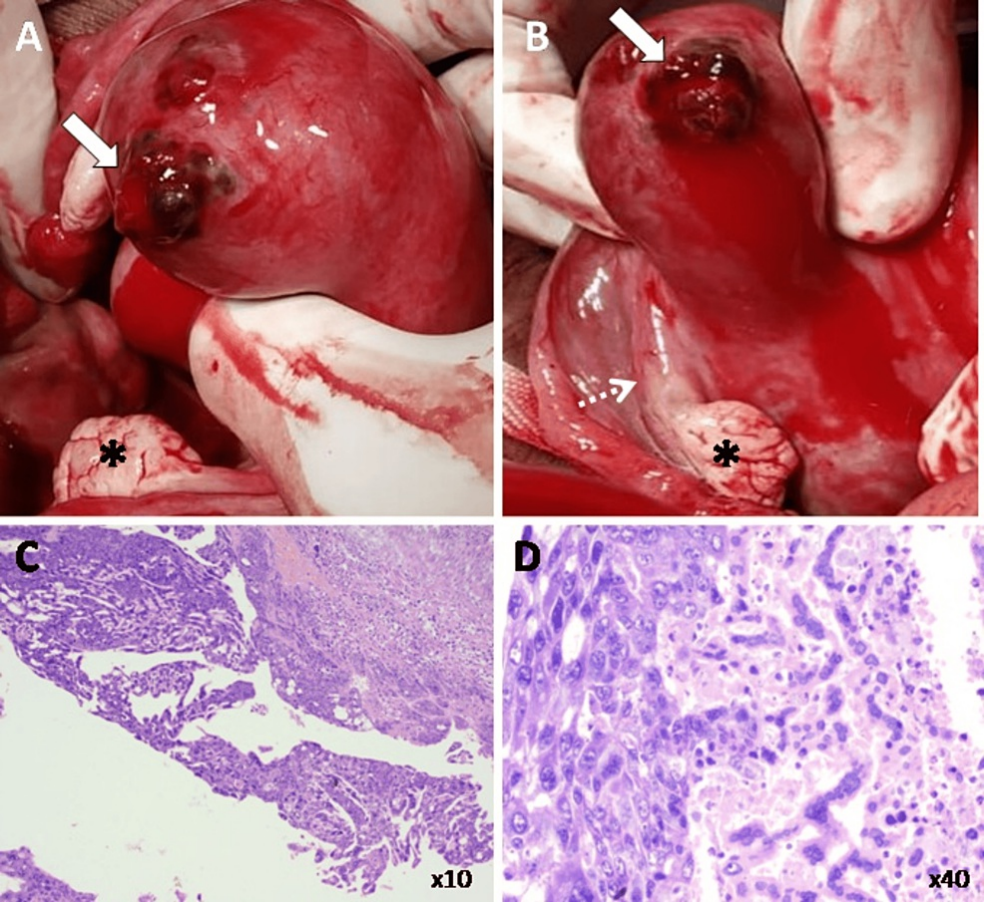
(A and B) Intra-operative photographs demonstrate a bicornuate uterus with a notable irregularity in the uterine profile and a pronounced bulge in the fundus of the rudimentary horn, accompanied by an associated uterine perforation (solid white arrows in A and B). The right cornu (dashed white arrow in B) and both ovaries (black asterisks in A and B) appear normal. Photomicrographs of a surgical specimen in Hematoxylin-eosin stain (C) at x10 magnification and (D) at x40 magnification show cytotrophoblasts and syncytiotrophoblasts with lymphovascular invasion.

**Figure 4:**
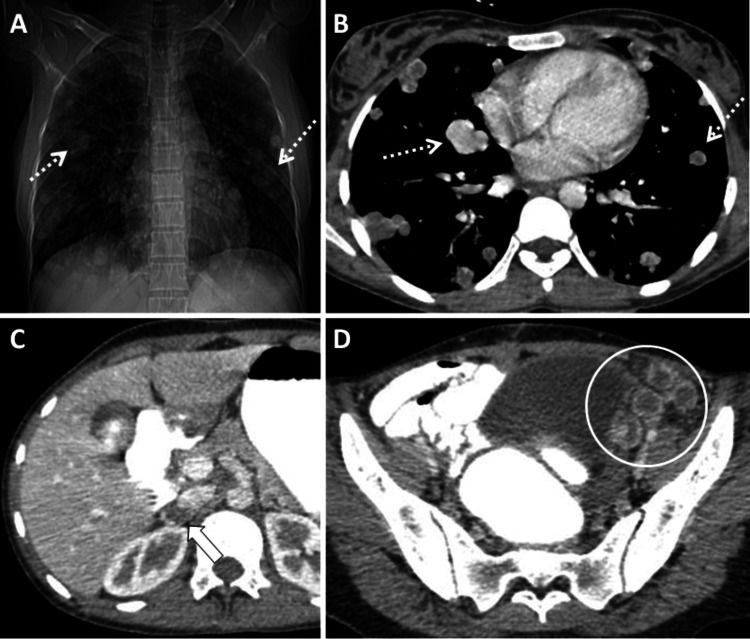
At two-month follow-up (A) chest radiography and (B) post-contrast CT chest show multiple lung parenchymal metastases with a typical cannonball appearance (dashed white arrows in A and B). (C and D) Post-contrast CT abdomen and pelvis demonstrate a right adrenal metastatic nodule (solid white arrow in C) and conglomerated necrotic metastatic deposits in the left lower quadrant along the external iliac chain (open white circle in C).

The patient subsequently underwent an emergency laparotomy, encompassing a total abdominal hysterectomy and bilateral salpingo-oophorectomy. Intra-operative observations unveiled a bicornuate uterus with a notable irregularity in the uterine profile and a pronounced bulge in the fundus of the rudimentary horn, accompanied by associated uterine perforation. The right cornu and both ovaries appeared normal (Figures 3A, 3B). The pelvis MRI did not distinctly represent the bicornuate anomaly as a non-uterine protocol was executed, given the emergent context and distortion of uterine contour by the mass lesion. The surgical procedure and the immediate post-surgical phase transpired without setbacks, and the patient was discharged in stable condition after that. Histopathological evaluations authenticated choriocarcinoma and lymphovascular invasion, culminating in a pathological classification of pT1 (FIGO stage 1) (Figures 3C, 3D). One month later, a follow-up serum β-hCG measurement again exceeded 100,000 IU. As a result, the patient was referred to the oncology division, where she began chemotherapy. Following three chemotherapy sessions over the following month, a chest radiograph and contrast-enhanced chest and abdomen computed tomography detected metastatic involvements in multiple pulmonary parenchymas, the right adrenal gland, and pelvic lymph nodes (Figure 4). Unfortunately, the patient was unable to survive due to widespread metastatic disease.

## Discussion

GTD encompasses a range of disorders originating from trophoblastic cells, spanning both benign and malignant manifestations [1]. While benign GTD includes partial hydatidiform and complete hydatidiform molar pregnancies, its malignant counterparts are an invasive mole, a placental site trophoblastic tumor, choriocarcinoma, and an epithelioid trophoblastic tumor. Specifically, an invasive mole, choriocarcinoma, and a hydatidiform mole arise from villous trophoblasts. Conversely, intermediate trophoblasts derive from the placental site trophoblastic tumor and epithelioid trophoblastic tumor [2].

The incidence of choriocarcinoma exhibits substantial variation worldwide. For instance, while the prevalence in the United States is relatively low at two per 100,000 pregnancies, figures escalate to 202 per 100,000 pregnancies in China [3]. Notably, choriocarcinoma represents the most aggressive GTD variant, characterized by its swift growth dynamics and significant metastatic potential. Typically, choriocarcinoma disseminates extensively via hematogenous pathways, often correlating with distant metastases. A history of molar pregnancy is a prime risk factor for choriocarcinoma [5], a criterion met in the present case.

The clinical presentation of choriocarcinoma can vary extensively, encompassing symptoms like irregular vaginal bleeding, nausea, vomiting, breathlessness, and headache. However, spontaneous uterine perforation emerging as the preliminary symptom is exceedingly rare [6]. A study by Xiang et al. evaluating 68 patients with trophoblastic neoplasia treated through hysterectomy from 1985 to 1997 identified 18 instances of uterine perforation [7]. Choriocarcinomas are inherently highly vascular tumors, synthesizing abundant angiogenic growth factors. Concurrently, there is pronounced cytotrophoblast proliferation, leading to the invasion and remodeling of blood vessels. Such neo-angiogenesis can precipitate infarctions, spawn neoplastic aneurysms, and induce hemorrhage/necrosis within the tumor, adjacent tissues, and myometrium. Potential ruptures of thrombosed spiral vessels might culminate in uterine rupture [6,7]. A study by Chan et al. underlined the rarity of choriocarcinomas triggering uterine rupture, consequently leading to hemoperitoneum [8]. Intriguingly, the current case's presentation mirrored this rare manifestation, marked by uterine perforation and hemoperitoneum.

Imaging serves as a pivotal tool for both the diagnosis and staging of choriocarcinoma. Ultrasonographically, choriocarcinoma can manifest as hyperechoic, hypoechoic, or heterogeneous myometrial mass, often blurring the demarcation with the endometrium and perimetrium. Notably, cystic regions within the myometrium can be discerned. Some masses also exhibit arteriovenous malformations, a manifestation of neo-angiogenesis. The cystic spaces may encompass hemorrhage, necrosis, cysts, or vascular channels. Discriminating choriocarcinoma from an invasive mole remains challenging. Color and spectral Doppler investigations supplement the choriocarcinoma diagnosis, displaying low impedance and resistance waveforms [9].

On MRI, choriocarcinomas present as heterogeneous masses-predominantly hypo- to isointense on T1 and hyperintense on T2W images with pronounced post-contrast enhancement. MRI delineates tumor morphology, gauging tumor volume, invasion depth, and local extent of malignant gestational trophoblastic tumors. Intra-tumoral hemorrhagic and necrotic zones are discernible. A hallmark MRI feature is the discernible enlarged flow voids encircling the mass, hinting at dilated and tortuous uterine and other myometrial arteries. MRI emerges as a superior modality for tumors ensconced within the myometrium or exclusively situated therein [10].

Meanwhile, CT primarily aids in gauging metastasis, which afflicts approximately 10-19% of patients with gestational trophoblastic neoplasia [10]. Predominantly, the lungs are the metastatic focal point, succeeded by the vagina, liver, and kidneys. In the presented case, the patient exhibited metastasis to both the lungs and the adrenal gland-a rather unusual presentation.

Literature on choriocarcinoma within a bicornuate uterus remains scant, with limited case reports accessible. Singhai et al. detailed a case of a patient who had been operated on (excision of a rudimentary horn) two years back for silent rupture of the rudimentary horn of the bicornuate uterus due to a perforating mole. The patient subsequently presented with four months of amenorrhea, and ultrasonography suggested an invasive mole or choriocarcinoma, with a subsequent hysterectomy confirming choriocarcinoma [11]. Kaplan et al. outlined a case involving an invasive mole within a unicornuate uterus and a communicating rudimentary horn. Post-laparoscopic resection of the rudimentary uterine horn, the β-hCG levels normalized within a month [12]. In another distinct report by Kulkarni et al., a molar pregnancy in a bicornuate uterus was managed via suction and evacuation, with declining β-hCG levels indicating remission [13]. Intriguingly, case reports spotlighting endometrial carcinoma in the bicornuate uterus outnumber those focusing on choriocarcinoma in a bicornuate uterus [14].

Despite an exhaustive review of the available literature, no reports were found documenting choriocarcinoma in a bicornuate uterus presenting with uterine perforation. Due to the aberrant morphology inherent to the bicornuate uterus, the malformed organ faces challenges in expansion as gestation progresses. This results in an abnormally attenuated uterine wall, predisposing to an escalated risk of rupture as gestation advances [15]. Such a mechanism is the plausible etiology behind the uterine perforation observed in the current patient.

## Conclusions

Choriocarcinoma in a bicornuate uterus culminating in uterine perforation remains an exceptional rarity. Ultrasonography is an essential modality for initial evaluation and primary identification, while MRI is instrumental in affirming the diagnosis, undertaking local staging, and identifying potential complications. CT, on the other hand, is pivotal for assessing metastatic spread. A timely and precise diagnosis of uterine perforation linked to choriocarcinoma is imperative to facilitate prompt surgical action. The case report findings underscore the necessity of vigilance in identifying uterine anomalies on imaging during early gestation or in instances of molar pregnancy to preempt and forestall complications in subsequent stages.
